# Humanising and dehumanising pigs in genomic and transplantation research

**DOI:** 10.1007/s40656-022-00545-4

**Published:** 2022-11-22

**Authors:** James W. E. Lowe

**Affiliations:** Science, Technology and Innovation Studies, University of Edinburgh, Old Surgeons’ Hall, High School Yards, Edinburgh, EH1 1LZ UK

**Keywords:** Genomics, Transplantation, Xenotransplantation, Comparative practices, Homology, Species boundaries

## Abstract

Biologists who work on the pig (*Sus scrofa*) take advantage of its similarity to humans by constructing the inferential and material means to traffic data, information and knowledge across the species barrier. Their research has been funded due to its perceived value for agriculture and medicine. Improving selective breeding practices, for instance, has been a driver of genomics research. The pig is also an animal model for biomedical research and practice, and is proposed as a source of organs for cross-species transplantation: xenotransplantation. Genomics research has informed transplantation biology, which has itself motivated developments in genomics. Both have generated models of correspondences between the genomes of pigs and humans. Concerning genomics, I detail how researchers traverse species boundaries to develop representations of the pig genome, alongside ensuring that such representations are sufficiently porcine. In transplantation biology, the representations of the genomes of humans and pigs are used to detect and investigate immunologically-pertinent differences between the two species. These key differences can then be removed, to ‘humanise’ donor pigs so that they can become a safe and effective source of organs. In both of these endeavours, there is a tension between practices that ‘humanise’ the pig (or representations thereof) through using resources from human genomics, and the need to ‘dehumanise’ the pig to maintain distinctions for legal, ethical and scientific reasons. This paper assesses the ways in which this tension has been managed, observing the differences between its realisations across comparative pig genomics and transplantation biology, and considering the consequences of this.

## Introduction

The physiology of the pig (*Sus scrofa*) is close enough to humans to make them valuable for toxicological testing, pharmacokinetic and pharmacodynamic studies, surgical research and training, and as a model of various diseases, such as cardiovascular conditions. Their utility as an animal model for humans has been used to justify funding pig genome research (Rohrer et al., 2002). Pigs are also a favoured potential source of non-human organs for xenotransplantation: the transplantation of cells, tissues and organs across species boundaries. Xenotransplantation has been explored as one possible solution to the shortages of organs available for transplantation.^1^

In common with other animal models and model organisms, for the pig to be of use for biomedical research, a set of tools and resources must be developed to establish how data and knowledge deriving from it can inform understandings of human biology, pathology and treatment. This paper discusses two aspects of this: research on pig genomics, and studies of porcine immunology and xenotransplantation. In both of these areas of research, there is an ongoing tension between the humanisation of the pig (or representations of it) and the dehumanisation necessary to ensure that the pig (and representations of it) remain sufficiently porcine. Due to the variant goals of these areas of research, and the distinct ways in which they must relate the pig to the human, the tension between humanisation and dehumanisation manifests in different ways.

Comparative genetics research, and the use of comparative methods in the creation and deployment of animal models, have a long history (e.g. Ankeny & Leonelli, 2011; Creager, 2002; Friese & Clarke, 2012; Nelson, 2013; von Schwerin, 2013). The comparison of genomic data belonging to different species is central to genomics research and biological research more broadly (García-Sancho, 2012; Strasser, 2019). To advance pig genomics, researchers have made use of “trans-species shuttling strategies” (after Georges & Andersson, 1996). These strategies have involved the identification and characterisation of ever more precise relations of homology – similarity – between the genomes of pigs and humans, thus allowing pig genome researchers to use the more plentiful resources of human genomics (including maps of genes and genetics variants, as well as DNA sequence data) to inform and improve their own representations of the pig genome. Researchers have identified and exploited inter-specific affinities to make use of data, knowledge, materials and practices from human genomics. They have also used their own species-specific knowledge of swine to ensure that the humanising potential of their methods was corrected by a dehumanisation of the genomic representation of the pig. In other words, they have had to maintain and monitor species boundaries while necessarily crossing them in the course of their work.

For xenotransplantation, researchers interrogate genomic, immunological and physiological differences between pigs and humans to identify ways in which ‘donor’ pigs can be humanised to reduce the likelihood of rejection of transplanted organs. The data and knowledge produced by comparative genomics has allowed xenotransplantation researchers to pinpoint precise immunologically-relevant genetic differences between pigs and humans. They can use this information to humanise the DNA and immune systems of pigs in highly-specific ways.^2^

Transgressing the species boundaries in transplantation biology depends upon the strict managing of species boundaries in comparative genomics. This is because one must first be clear about what is specific and distinct about the genomes of pigs and humans, in order to identify and verify the differences between the species that constitute barriers to successful cross-species transplantation. This means that researchers need to pay close attention to the ways that the data, representations, methods and materials derived from human genomics are used, to prevent representations of the pig genome and its contents being humanised in a way that would obscure efforts to discern genetic differences between the two species. A different mode of policing the distinction between pigs and humans is present in xenotransplantation: the pigs may be humanised, but however humanised they become, they must still remain pigs, as it is their non-human (and, indeed, non-primate) nature that makes them worthwhile candidates for this role.

In sections two and three, I examine these areas of research in turn, to explore how the tension between humanisation and dehumanisation operated differently in them. In section two, I provide some historical and theoretical background on cross-species comparative practices in pig genomics, and investigate these in more detail with a study of the development and use of a radiation hybrid panel. This was constructed to provide maps of the pig genome at increasingly higher resolutions. Genome maps are representations of the relative – and in some cases, absolute – positions of elements such as genes on the chromosomes of particular species. I conclude section two with a reflection on the nature of these comparative practices, in terms of models and modelling, and as an epistemic activity.

In section three, I inspect transplantation biology by outlining the research agenda of a key institution, the ‘Laboratoire Mixte CEA-INRA de Radiobiologie Appliquée’ (hereafter ‘CEA-INRA’), over four decades. CEA-INRA investigated first the genetics and then the genomics of pig immune response biology, to develop the pig as a model for transplantation research. This work also fed into research on xenotransplantation from the 1990s onwards. CEA-INRA therefore constitutes a key connection between the two manifestations of the tension outlined in this paper. Following an account of how xenotransplantation researchers have used and developed comparative genomics to discern key differences between pigs and humans to effect a limited – but more literal – humanisation of the pig, I conclude section three with an examination of the particularities of the analogous tension between humanisation and dehumanisation as it is manifested in genomics and transplantation research.

In the final section of the paper, I consider how my findings might be situated and extended in the wider biological sciences beyond the limited aspects of the dyadic human-pig relationship covered here.

## Managing the tension between humanisation and dehumanisation in pig genomics

The first serious efforts to map the pig genome began in 1989.^3^ The potential for using the mapped genes and genetic markers of human and mouse in the mapping of the genomes of other mammals was recognised from the advent of genome mapping projects. For instance, in their research proposal to the European Commission, the consortium wishing to establish a Pig Gene Mapping Project (PiGMaP) claimed that “[t]he comparative conservation of DNA sequences and linkage relationships between mammalian species allows the porcine genetic map to be built on a skeleton of molecular markers which have already been mapped in man or in the mouse”.^4^

However, rather than simply providing an initial skeleton onto which pig-specific data would provide the flesh in future, once maps were sufficiently developed to enable comparisons between the genomes of different species to be made, a cumulative iterative process was established. The identification of ever more precise patterns of correspondence between parts of the genomes of different species enabled maps to be populated with progressively more markers. This, in turn, aided the further elaboration of inferential relations of homology between areas of the richly-populated map of the human genome and the more underpopulated representation of the pig genome.^5^ In this process, new datapoints were added to an existing map; these then constituted a foundation on which novel representations of the genome could be constructed. For instance, existing markers on a linear map of a given chromosome were used by researchers to add new markers to the map, by triangulating between them. This also enabled the production of new maps, such as comparative maps between species, like the one discussed in Sect. 2.2.

PiGMaP and similar projects were contemporary to efforts to map the genomes of other species. Accompanying this was an effort to establish a conceptual and infrastructural basis for the pursuit of comparative genomics.^6^ This drive was originally pioneered in mouse genetics and later spread to other mammals (Womack, 1987).^7^ In order to advance it, researchers devised criteria for discerning and justifying inter-species homologies between genomes, identified corresponding blocks of evolutionarily conserved genomic regions, created databases to aid the identification and recording of homologies, and developed species-specific resources and maps to help with the comparative mapping and inferences. These form part of what I term *models of correspondence*, comparative complexes of models comprising of similarity relations between materially and intellectually abstracted representations (after Leonelli, 2008) of the genomes of two different species. Attributions of comparability or correspondence between species were justified by establishing the existence of relevant similarities or homologies (Brigandt & Griffiths, 2007). The inferential apparatus was then used to populate maps of the genomes of species like pigs with more and more genes and genetic markers, with increasing levels of precision and accuracy. In turn, this process also refined the inferential apparatus itself. One way in which this apparatus has been shaped by communities of geneticists has been to try to prevent data and resources derived from other species (such as humans) from being merely *transferred* into the maps of target genomes, in which they may not belong.

### Laying the foundations for comparative genomic strategies

To advance pig genomics, concepts, definitions and standards that could be employed across species needed to be articulated and deployed in new infrastructures. This required a common endeavour beyond individual species-centred communities, and this was indeed evident in non-human mammalian genomics (Womack, 1987).

Conceptually, the most significant developments in the early-1990s were the elaboration of the significance of the conservatism of mammalian genome evolution; the classification of genetic markers into two types, to highlight those that would be instructive in making cross-species inferences; and the establishment of a common language of kinds of inter-species homologies and criteria for discerning them.

In comparative genomics, conservatism means that some sections of human chromosomes correspond or are homologous (similar due to common ancestry) to parts of the chromosomes of other species. These homologous regions could be discerned through identifying genes and markers mapped to the genomes of non-human mammals that were homologous to some mapped human gene or marker. The initial mapping of genes and markers therefore allowed the detection of conserved elements of the genome across species. This allowed researchers to infer the presence and location of additional markers and genes that could then be identified and mapped, thus aiding the further delineation of conservation across species. These comparisons are cumulative and iterative. They also enable inferences concerning genomic re-arrangements to be drawn that help to explain observed patterns of correspondence, and thus contribute to new knowledge about the evolution of the genome. Although the ability to identify and map homologous genes and markers across species provided a considerable motor for this work, cytogenetic analysis allowed the initial comparison of chromosomes across species on a gross scale, and aided the physical mapping of particular loci (the locations of genes or markers on chromosomes). Physical mapping means determining the physical distances between loci, rather than the relative positions produced by linkage or genetic mapping.

The significance of conservation and homology for developing genome mapping was elaborated in a 1991 paper by geneticist Stephen J. O’Brien, whose conceptual work was cited in many pig genome mapping publications. In this paper, O’Brien classified genetic markers into two types: Type I denoting “anchor loci [that] are evolutionarily conserved, coding genes”, and Type II designating “species-specific DNA markers that exhibit a high degree of polymorphism.” O’Brien noted that early mapping efforts on humans and mice had begun with Type I mapping, but that due to the difficulties with this, newer mapping efforts would likely begin with Type II mapping, which he believed would make comparative approaches tricky. However, using Type I loci to anchor the mapping of the more abundant and easily-identified Type II loci would enable the adoption and advancement of comparative genomic methods, while exploiting intra-specific genomic variation to “complement the skeletal framework provided by the type I map” (O’Brien, 1991, pp. 108–109; note the similarity to the formulation used by the PiGMaP promoters). O’Brien’s cautious language belied the extent to which comparative approaches would power the development of genomic data and resources.

Many pig genome researchers attended The First International Workshop on Comparative Genome Organization (TFIWCGO) in 1995, which featured discussions of criteria for determining inter-species homologies and the need for standard nomenclature and appropriate informatics infrastructure to enable comparative mapping. Many of the criteria for assessing the homology of markers discussed at this workshop derived from reports of the Comparative Gene Mapping Committee of prior human gene mapping workshops, with O’Brien the lead reporter of these in the early-1990s. They recommended that evidence for homologies be recorded on The Comparative Animal Genome database, hosted and managed at the Roslin Institute, a key site of livestock genetics mapping and informatics (Lowe, 2021). A key source of information cited in the TFIWCGO report was the Mouse Genome Database, managed by the Jackson Laboratories, which featured homologies of “specific chromosomal regions, gene symbols, gene names, and citations” across over 50 mammals, including the pig (TFIWCGO, 1996). The advent of data infrastructures like these are also part of what enable model organisms to perform their function as a comparative tool (Leonelli & Ankeny, 2012).

Contemporary with these efforts to establish a conceptual and infrastructural basis for comparative genomics, was an increasing empirical corpus that was informing the models of correspondence being conceived between the genomes of human and non-human animals. For instance, a 1996 review by Michel Georges and Leif Andersson noted that pig genome mapping research had complicated the assumptions concerning the finer details of genomic conservation between human and other mammalian genomes. Most significantly, this research found that in areas of *conserved synteny* where the same two homologous genes are present in both humans and another mammalian species, these genes do not always lie in the same order and relation to each other on the chromosome. Georges and Andersson concluded from this that higher-resolution comparative mapping to identify the relative positions and orders of the genes (or other genomic elements) *within* a block of conserved synteny between humans and other mammals was required for cross-species inferential purposes.

At the time of this review, the increasing availability of mapped human protein-coding DNA sequence (especially Expressed Sequence Tags, ESTs) was of interest to livestock geneticists: they were Type I markers and therefore likely to be conserved across species. To move beyond a reliance on conserved synteny and the limited resolution of methods such as linkage mapping and bidirectional chromosomal painting by Fluorescence in situ hybridization or ‘FISH’ (on each, see Lowe & Bruce, 2019), Georges and Andersson advocated “the development of efficient ‘trans-species shuttling’ strategies” to properly exploit the human genomic bounty. These strategies including increased mapping of coding sequences to improve the resolution of comparative maps, which would enable the use of human ESTs in livestock genomics. Georges and Andersson suggested the development of radiation hybrid (RH) panels and DNA libraries (in which cloned fragments of the DNA of a species could be stored) to advance this (Georges & Andersson, 1996, quotation from p. 916). Both would indeed be pursued to execute the trans-species shuttling strategies they recommended. The purpose of RH mapping is discussed below in connection with its role in comparative mapping and the whole-genome pig physical mapping and DNA sequencing projects.

The follow-up comparative gene mapping workshop in 1999 reported considerable progress on the use of human ESTs for identifying and mapping homologies in other species – including the pig – as well as the development of EST collections for non-human animals (Gellin et al., 2000). Both were used in the construction of RH maps, using RH panels produced for the purpose, illustrating the species-specific work that must take place for the furtherance of the material, methodological and theoretical infrastructure for making comparative inferences across species.

### Radiation hybrid panels and comparative mapping

RH mapping was originally conceived as a method for mapping genes in the 1970s (Goss & Harris, 1975), and was implemented for mammalian genome mapping in 1990 (Cox et al., 1990). The basic approach is to irradiate chromosomes using X-rays, to break them apart into multiple fragments.^8^ From this, the co-retention of specific genetic markers can be identified. The frequency at which two (or more) markers can be detected on the same fragment provides a means to identify how closely linked the markers are on the chromosome. Statistical and computational procedures can be employed to calculate the likelihood of particular orders of markers based on the empirical data, and the results of this can be validated by cross-referencing to the findings of other physical mapping methods.

RH mapping requires expertise in the techniques deployed in it, and the identification of a set of markers that can be used. Once adopted, however, it becomes a powerful iterative mapping method, aiding the integration of Type I and II markers onto single maps and improving the resolutions of these and other maps.

One major effort towards pig RH mapping was made by a collaboration between a team from a station of the French national agronomic research institute (INRA) at Castanet-Tolosan near Toulouse and one at the University of Minnesota. They created the INRA-Minnesota porcine Radiation Hybrid (IMpRH) panel containing fragments of porcine DNA (Yerle et al., 1998). This was made available for the wider community to use in mapping, once the creators tested it using 903 markers deriving from previous mapping efforts. This allowed them to produce an RH map consisting of 757 markers in 128 linkage groups, including 19 ESTs and 39 genes (Hawken et al., 1999).

The potential of this resource for pig-human comparative mapping soon materialised. In 2002, another Franco-American collaboration mapped 1058 porcine ESTs using the IMpRH panel. They then compared the sequences of the porcine ESTs to the most up-to-date version of the human genome, to identify homologies.^9^ This had two main results. Firstly, it allowed them to assess and further refine prior conclusions concerning pig-human homologies and the history of changes to the genomes across evolutionary time. Secondly, it enabled the team to produce a pig-human comparative map using the homologous ESTs, the human reference genome sequence and existing knowledge concerning the location of blocks of conserved synteny (Rink et al., 2002).

That same year, the expanded Franco-American collaboration produced a new RH panel: IMNpRH2.^10^ This new panel was constructed on the basis of a 12,000-rad dose of radiation, higher than the 7,000-rad IMpRH (Yerle et al., 2002). Generally, the higher the dosage of radiation, the smaller the resulting fragments of DNA. This improves mapping resolution, as markers co-retained on smaller fragments of DNA are more likely, *ceterus paribus*, to be physically closer than markers co-retained on larger fragments. The new panel was intended to complement and build upon the older one, making use of its mapped markers, but requiring a higher number and density of markers in order to be operable. Until these were available, it could be used to increase the resolution of mapping and comparative mapping in regions already well-populated with markers.

Once sufficient markers became available, the cross-Atlantic collaborators could further the comparative project at both gross and fine levels. An example of the latter was the refinement of the delineated areas of conserved synteny in small corresponding regions of the human and pig genomes (Liu et al., 2005). For the former, they could use the IMNpRH2 panel to construct a pig-human comparative map, using ESTs and sequences of several hundreds of nucleotide bases at the end of DNA fragments stored in Bacterial Artificial Chromosomes (BAC; those stretches of nucleotide bases are known as BAC End-Sequences, or BES). Bacterial Artificial Chromosomes are a kind of DNA library in which cloned fragments of DNA are stored in artificial chromosomes inserted into the bacteria *Escherichia coli*.

**Fig. 1:**
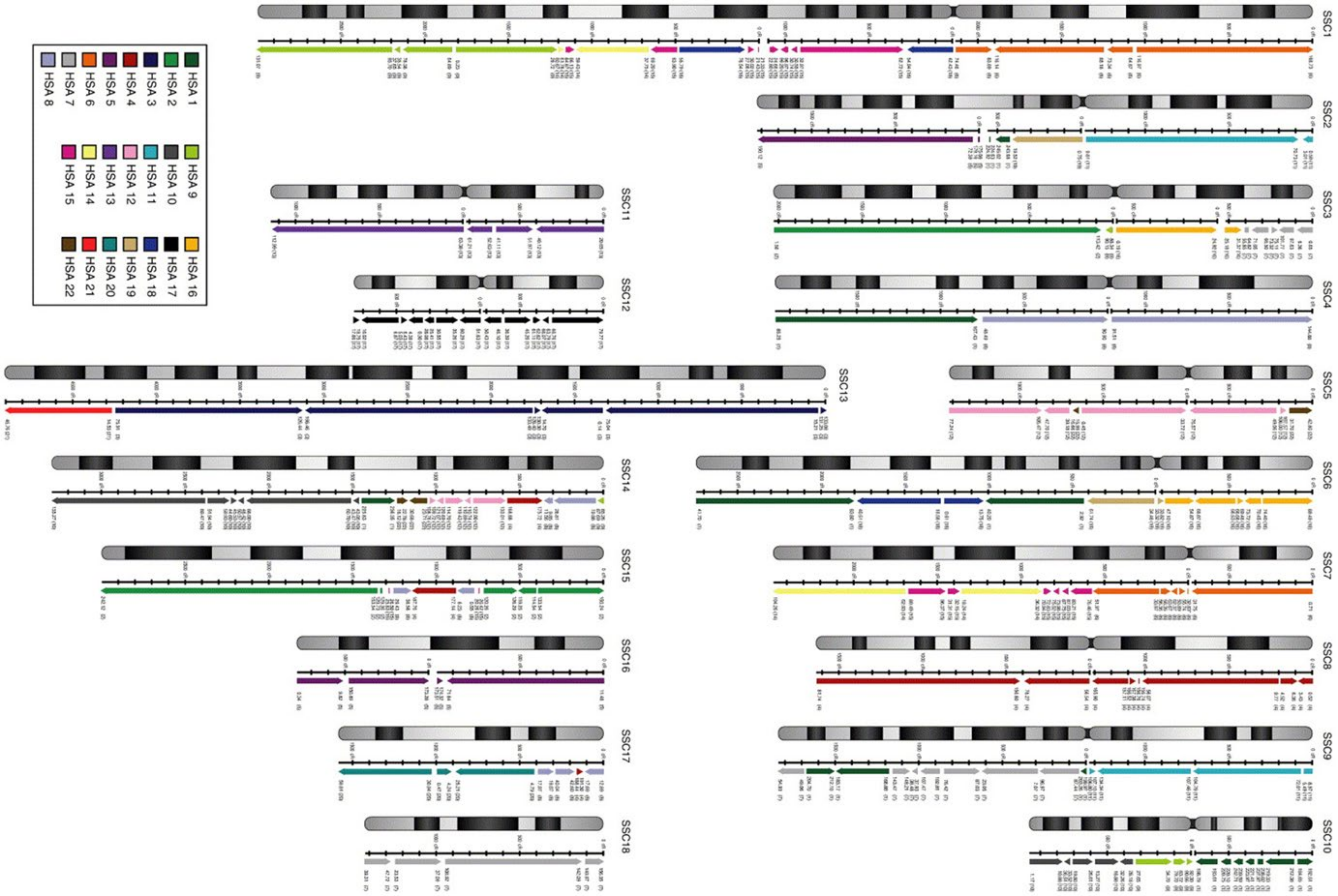
Pig-human comparative map produced using radiation hybrid mapping. Against the grey-scale pig chromosomes are aligned the corresponding regions of colour-coded human chromosomes. From Meyers et al. (2005).

The BES were chosen according to their similarity to human sequence, and to ensure a spread across the genome. The BES had the advantage of enabling mapping in non-coding regions of the chromosomes that were ill-served by ESTs. The comparative map so produced confirmed and extended knowledge of the identity and location of conserved regions. The visualisation of it arrayed the corresponding parts and orientations of colour-coded human chromosomes alongside grey-scale representations of pig chromosomes (Meyers et al., 2005; see Fig. 1). In so doing, it constituted a resource that could be used to identify potential porcine genes using human genome data, as well as aiding ever finer-grained mapping of markers, further comparative mapping, and the production of a complete physical map of the pig genome.

To achieve the latter, a comparative “strategy of piggy-BACing the human genome” was developed, in which BES would be matched to human genomic sequences using BLAST comparisons. BLAST, an acronym of Basic Local Alignment Search Tool, uses algorithms to compare DNA (and other nucleotide and protein) sequences and identifies matches above given thresholds (a practice with a long history before BLAST, see Stevens, 2011). Porcine BES that satisfied the criteria of unique matches to a particular position in the human genome were selected and used in RH mapping for a pilot effort between swine chromosome 13 and parts of human chromosomes 3 and 21 (Rogatcheva et al., 2008, building on research conveyed at a conference in 2004).^11^

Based on this, a complete physical map of the pig genome was produced using BES aligned to version 35 of the human genome sequence, with RH mapping data used to exploit pig-human homology still further.^12^ This enabled additional investigation of points of homology, as well as forming the basis for the mapping. The BAC clones were then ‘fingerprinted’ to identify overlaps between the clones.^13^ An automated computer programme used this fingerprint data to join fragments together into larger fragments, called contigs. An initially stringent threshold allowed the merging of these into over 12,000 contigs. Contigs with DNA sequence overlaps detected between them were then joined together if there was evidence to support this from the alignment of the potentially overlapping BES with the corresponding parts of the human genome sequence, based on pre-existing knowledge of the homologies between different regions of the genomes. This use of the human genome data to merge contigs was only permitted when the fingerprints overlapped. Otherwise, as the paper reporting the physical mapping noted, there was a risk of “‘humanizing’ the map assembly” (Humphray et al., 2007, p. 4). Following this, further mergers of contigs were effected using the RH map discussed above. In all, this considerably reduced the total number of contigs to just 172, and provided further data for fine-grained mapping of conserved regions. The smaller number of contigs could now be arrayed onto the RH map, resulting in widespread coverage across the genome. As the known contigs were now able to cover most of the genome, the smallest number of clones that needed to be sequenced to cover the whole genome could now be identified: a minimum tile path (Humphray et al., 2007).

Both the RH comparative map and the physical map it helped to produce also played a further role in the completion of the reference genome sequence. Alan Archibald, a leading pig genome mapper, used the comparative map to see if the draft sequence produced by the Sanger Institute matched what one would expect based on human-pig homologies, shuttling between the online genome browser displaying the latest version of the human genome, and the browser depicting the draft pig genome. For a recent version of the pig reference genome (released in 2017), he used the comparative map to direct him to corresponding well-characterised human genome regions that provided him with clues as to the causes of gaps that remained in the pig sequence. He stressed to me, however, echoing the words of the physical mapping paper, that as “you don’t want to produce a humanised pig genome,” the use of such comparative resources – either directly or as a shuttle between species – always had to be backed up by pig-specific data.^14^ Agreement with pig-specific data was also vital in the annotation of the pig genome, the identification and recording of particular genomic features, which also used BLAST to interrogate protein sequences of other vertebrates (Groenen et al., 2012, supplementary information).

The RH-derived comparative map has not been made obsolete by the production of a whole-genome physical map or a reference genome.^15^ It continues to function as a means to move between the material and intellectual abstractions of different species for proximate purposes such as finding homologous genes, and it therefore enables models of correspondence to be further developed.^16^

### Characterising trans-species shuttling strategies as comparative practices

The community of pig genomics researchers have endeavoured to ensure that in the development and execution of trans-species shuttling strategies, they use their existing knowledge of the pig genome to use human genomic data and materials indicatively and for sense-checking, and not to merely copy it over into the genomic maps and sequences of *Sus scrofa*. This managing of the tension between humanisation and dehumanisation has been vital in ensuring the production of a sufficiently porcine pig genome.

The models of correspondence between human and pig genomes are both material and formal *analogical models*, in that the pig and human genomes have observable properties in common and “the relations between certain elements within one domain are identical, or at least comparable, to the relations between corresponding elements in some other domain” (Bailer-Jones, 2009, p. 57, paraphrasing Mary Hesse). Understood as an analytical category, models of correspondence incorporate a variety of models of different kinds. These include: one-dimensional representations of chromosomes with labelled markers; mental models of chromosomal dynamics; computational or paper models of the relative orientation of sequenced fragments of DNA; and digital representations of the genomes of the two species.^17^

In their ongoing operation as mediating instruments (Morrison & Morgan, 1999), the models of correspondence are themselves continually developed, meaning that the processes of construction and use of these models are inextricably linked and simultaneous. Their main function is to enable movement between abstractions of models of different species’ genomes. They embody correspondences between genomic regions deemed equivalent according to criteria of homology established by comparative geneticists, such as those we encountered in Sect. 2.1. Additionally, they include more abstract relations and patterns of correspondence drawn from empirical data on the correspondence of concrete regions at varying scales. Through correspondences between the species being iteratively and recursively delineated, with comparative work producing more empirical evidence upon which to base further comparisons, a “consilience between observations in one species and those in another” is thereby produced (Griffiths, 2007).

The models and the modelling activity in the space between the human and pig enables the movement of data, expertise, methods and materials between them. This phenomenon is captured by Friese and Clarke’s articulation of “transposition”, which “directs analytic attention to the ways in which models create dynamic and iterative connections between different kinds of things and organizational sites.” Transposition, in this sense, concerns the moving of knowledge and things, and dynamic relationships between them (Friese & Clarke, 2012). This describes well the way that the trans-specific models discussed in this paper operate. They do not, however, mediate (after Morrison & Morgan, 1999) between theory and ‘the world’, but between existing representations (e.g. maps and sequences) and abstractions (be they material or otherwise) constituting their own sets of linked models characteristic of particular species: species domains.

The “dynamic relationships” incorporated in the transposition across species, mediated by inferential models of correspondence, are realised in the iterativity of connections between different species domains (e.g. concerning the genomes of pig and human). This iterativity is also cumulative, and can be understood as a form of *epistemic* iterativity, “in which successive stages of knowledge, each building on the preceding one, are created in order to enhance the achievement of certain epistemic goals” (Chang, 2004). This *mediating* iterativity, understood as transposition, captures the processes by which epistemic iterativity is achieved. This process of epistemic iterativity is manifested through continual testing and refinement of the models of correspondences between the genomes of the two species, as well as the development of the criteria of homology. The informatics, nomenclatural and social elements – discussed in Sect. 2.1 – that enable this work to be performed, must also be in place.

The articulation of models of correspondence, that enable mediation between species domains to achieve the iterativity related above, is crucial to understanding the nature and outcomes of such models, modelling and ‘trans-species shuttling.’ These models are themselves empirical and epistemic contributions to biological research, of potential use for researchers working with other species. Experiments can be conducted on pigs that would not be allowed on humans, and these generate data and knowledge that could then, through the very means of inference developed to make use of the resources of human genomics, be used to aid human geneticists and genomics researchers. For example, a mutation in the *RN* gene was discovered in pigs, and linked by researchers to high glycogen content. From this, the corresponding mutation in humans was identified, and found to have a role in non-insulin dependent diabetes (Costford et al., 2007). To develop the pig as a model for the human, though, it has been necessary to use the human as a model for the pig. Crucially, within the domain of each species, genomic resources, references and data themselves must still be *constructed*, not merely taken or borrowed. The interpolation of models functioning in the way described above is vital to appreciating this. The pig genome was constructed using inferential comparative models of correspondence; parts of the human genome were not merely copied across. These models work between species domains, are open-ended and indefinitely iterative, and result in the production of reference resources in genomics.^18^ Once constructed, the inferential function is both symmetrical and reciprocal.^19^

The inferential models themselves provide the means by which human genomic data and knowledge function as a model or resource. Production of new genomic data and knowledge on the pig was mediated by pig-specific knowledge concerning the biology of that organism, and the generation and use of pig specific materials (such as the ESTs) and data (for example on genetic linkage). Here, the potential for humanisation lurks within the inferential models. Through these lie the conduit for importing data and materials that, if passively accepted and applied to the pig genome, would humanise it by virtue of selectively adding new data concerning homologous genomic features, at the expense of neglecting the deepening of datasets and knowledge concerning non-homologous areas. The dehumanisation comes from the filtering provided by confirmation procedures using pig-specific data, and the generation of data to fill out the non-homologous regions. This may involve, for instance, using the Type II loci discussed above, or the identification of many thousands of single nucleotide polymorphisms in the wake of the genome sequencing project. We therefore observe a tension between harnessing sufficient humanisation of the pig to produce genomic resources, and a corrective dehumanisation. In the next section, an alternative manifestation of the tension between humanisation and dehumanisation is articulated in the context of transplantation biology and xenotransplantation research, which makes use of the data and inferential architecture of genomics but features different epistemic goals and ways in which the pig operates as a model.

## Humanisation and dehumanisation at the intersection of genomics and xenotransplantation research

Prior research has established CEA-INRA as a key institution at the nexus of immunological and pig genomics research (Lowe et al., 2022). Here, it serves as an illustrative link from the comparative genomic tension between the humanisation and dehumanisation of pigs articulated above, to the way it operates in transplantation research.^20^ In this section, I begin by discussing how a team at CEA-INRA advanced their transplantation research through the adoption of genomic methods and the creation of genomic data and resources. Beginning in the 1960s, the main focus of their programme was allotransplantation, the transplantation of organs and tissues between genetically-different individuals of the same species. Although xenotransplantation was therefore not a central focus of theirs, the genomic and immunological resources and knowledge they produced through their work was a vital grounding for xenotransplantation research, which flowered from the 1990s onwards.

As well as the resources produced at CEA-INRA, populations of miniature pigs with well-characterised immune genetics had been established by other groups to further allotransplantation research. By the 1990s, it had been established that pigs were sufficiently anatomically and physiologically similar to humans to be promising candidates for xenotransplantation, even if their immune systems were understood to be quite distinct (e.g. Kenmochi et al., 1994; for a general history, see Deschamps et al., 2005). Pigs are phylogenetically more distant to humans than non-human primates are, but are more abundantly available, are prolific, their breeding can be controlled, and as a farm animal they are deemed to be a more acceptable subject of this kind of research by many more people.^21^

This, and the advancement of transgenics as a potential method for combatting immune rejection, was enough to prompt a flurry of research and investment concerning pig-to-human transplants, peaking from c.1992 to c.2001. The failure to overcome immune rejection of the organs originating from a different species and the increasing perception of risks to the enterprise – viral, regulatory, ethical – led to the cessation of private funding.^22^ In recent years, the advent of more accessible genomic sequencing and genome editing has led to the re-emergence of xenotransplantation as a promising solution to growing transplantation waiting lists (e.g. Editorial, 2016), as part of a range of measures to re-engineer – to humanise – the pig to make it a more reliable, safe and effective source of organs and tissues (Sykes & Sachs, 2019). The use of genomic data and resources on the pig has been vital to this endeavour. Much of this has been based on the foundations of comparative approaches to genomics that harness models of correspondences between the genomes of the pig and human. Below, I detail how these approaches have been pursued in the more specialised domain of immune response genetics, as a gateway to first advancing transplantation biology, and then the project of xenotransplantation.

### From allotransplantation models to xenotransplantation: genetics and genomics research at CEA-INRA

A highly collaborative institution from its inception in 1964, CEA-INRA was originally funded by both CEA, the French atomic energy agency, and INRA, the French national agronomic research institute. It was located on the INRA campus in Jouy-en-Josas, near Paris.^23^ The initial remit of the group established under the leadership of Marcel Vaiman was radiobiological, to use pigs as a model to understand the biology of bone marrow transplantation, to help restore bone marrow killed through irradiation.^24^

Initially, they performed autologous transplantations, in which the pigs’ own bone marrow cells were extracted before irradiation, and then replaced post-irradiation. This procedure was successful. Following this, they tried to make allografts work, transplanting bone marrow cells between genetically-different individuals of the same species. They then worked on skin grafts. Neither of these produced the same success as autologous transplantation. Using the blood products of immune responses, they started producing antiserum, developing many sera through conducting skin grafts between pigs in the same family. With this serological bank they could immunologically-type pigs, at least within families.

In 1970, CEA-INRA researchers demonstrated that pigs as well as humans possess a densely-packed set of highly-variable genes called the Major Histocompatibility Complex (MHC). In humans this is called the Human Leucocyte Antigen complex (HLA, see Thorsby, 2009), and in pigs the Swine Leucocyte Antigen complex (SLA). The team at CEA-INRA had been aided by collaboration with Jean Dausset and his team since 1968, who had discovered the HLA complex.^25^

Some of these MHC genes code for proteins implicated in immune response, such as those involved in the presentation of antigens at the surface of cells. Circulating immune response cells called T leucocytes will initiate an immune response if they encounter an antigen not derived from that individual’s MHC. The variation in MHC genes underpins the ability of immune systems to distinguish between self and non-self; simply put, the closeness of a particular set of MHC variants between two individuals determines how immunologically compatible they are, and so any immune rejection of a graft between them will be less intense and/or immediate. At this time, serological methods were needed to infer the existence of variants of MHC genes, and to therefore establish different sets of MHC variants with distinct serotypical profiles: these sets of particular variants are known as haplotypes. With the identification of particular haplotypes, the compatibility of the pigs could be assured for experimental allografts, enabling surgeons to use these pigs to hone and pioneer techniques for human allotransplantation.

Throughout the 1970 and 1980s, CEA-INRA continued to map the SLA. Due to its densely-packed and highly-polymorphic nature, they were eager to adopt new genomic methods for mapping it and characterising haplotypes. An early example of this is another fruit of the laboratory’s links with human geneticists: the use of human DNA probes from Dausset’s Centre d’Etude du Polymorphisme Humain (Geffrotin et al., 1984).

From the mid-1980s, the team augmented their serotyping by adopting molecular genetics methods to identify specific SLA loci and discern polymorphisms in an increasingly detailed way. In the 1990s, the CEA-INRA group became involved in efforts to systematically map the pig genome, joining PiGMaP to further develop their genomic focus on the SLA. This allowed more fine-grained analysis of the complex than serotyping could.^26^ At the outset of PiGMaP I (1991–1994), they produced probes to identify coding sequences in the SLA, to help find quantitative trait loci, sites in the genome associated with variation in a phenotypic trait.^27^ From this basis, they were able to physically map particular genes.

To further refine this physical mapping work, CEA-INRA created a genome library in Yeast Artificial Chromosomes (YACs). This line of work began in 1994, and the library was ready for use in 1997.^28^ Subsequently, they developed a BAC library.^29^ This work, as with their preceding research, was intended to aid the identification and mapping of SLA genes. Although the aim of the mapping projects themselves were to develop maps that were fairly densely-populated with markers, they were not densely-populated enough for the sort of work that CEA-INRA were pursuing. A comprehensive map was required for this, and this was constructed from 2002 to 2005, in part using the INRA BAC library. The CEA-INRA team also explored the libraries they created to clone the SLA and improve their knowledge of the complex.

It is here where CEA-INRA’s programme of research on the SLA, inspired by the problem of allotransplantation, intersected with the xenotransplantation research effort. Their first published use of the BAC library, in 1999, involved screening it to see if they could detect signatures of the presence of Porcine Endogenous Retroviruses (PERVs) in pig DNA (Rogel-Gaillard et al., 1999). Concerns had been raised about the presence of PERVs in 1997 (Le Tissier et al., 1997; Patience et al., 1997; the potential problem had been raised by Stoye & Coffin, 1995), following promising progress on combatting the rejection of transplanted pig organs in non-human primates in the preceding years (Cozzi & White, 1995; Sharma et al., 1996). The potential for infection of human transplant recipients from activated PERVs embedded in pig DNA was especially problematic given the immunosuppression induced in such patients to prevent graft rejection. That such infection had only been demonstrated in cell culture was not sufficient to allay concerns. Once the alarm had been raised about PERVs, research began to be conducted on their presence and prevalence in pig DNA, and whether they could pose a threat to human recipients of pig organs (e.g. Denner, 2016).

The genomic basis for such studies was the panoply of resources developed for the pig through the comparative approaches discussed above (and in Rogel-Gaillard et al., 1999). In addition, the annotation of the swine reference genome involved the identification of PERV sequences in the DNA sequence that was delineated (Groenen et al., 2012, supplementary information). Genomics therefore had much to contribute towards the examination of PERVs; conversely, the need to evaluate the risks potentially posed by PERVs shaped the direction of genomic research. The work on PERVs generated considerable information about the nature and history of the virus, and presented the possibility of creating pigs free of PERVs, or at least with a low risk of retroviral activation. One potential way to do this is to use genome editing to remove the retroviruses from the genome (Yang et al., 2015; Niu et al., 2017).

In addition to screening the BAC library for signatures of PERVs, CEA-INRA pursued the sequencing of ever larger genomic regions of interest. Some of this work was explicitly framed in terms of xenotransplantation research. For example, in 2001 they discussed the sequencing of 158,063 nucleotides with the aim of identifying porcine antigens that may trigger a human immune response and different kinds of graft rejection processes (Chardon et al., 2001). This work, on the class I set of SLA genes, continued up to 2005. This was incorporated into the overall sequencing of the SLA, completed later in 2005. While the class I genes had been sequenced with the aid of the French national sequencing centre Genoscope, sequencing of the class II and III regions took place at the Wellcome Trust Sanger Institute, which had already embarked on the physical mapping of the whole pig genome, and was to conduct the bulk of the whole-genome sequencing. CEA-INRA’s Christine Renard and the Sanger Institute’s Human And Vertebrate Analysis and Annotation (HAVANA) team worked to identify and record genes and other genomic elements. A key feature of the paper announcing the completed sequencing was a comparison with the HLA (Renard et al., 2006).

CEA-INRA’s main programme of research concerned allotransplantation. This work constituted a link between xenotransplantation and genomics. Two main problems for xenotransplantation are the potential for retroviruses embedded in the DNA of pigs to be activated in human transplant recipients, and rejection of the transplanted organ. CEA-INRA’s work encompassing the realms of comparative genomics and transplantation biology formed a platform for the research that aimed to combat these problems.

### Combatting rejection

Immune rejection comes in three levels of increasing onset time and difficulty to tackle: hyperacute, acute and chronic.^30^ Hyperacute rejection is an immediate rejection of the donor organ by the recipient’s body. Acute rejection occurs between days and a few months after transplantation, while chronic rejection is a longer-term process over the course of years. At CEA-INRA, their programme of research aiming to understand the nature of allotransplantation graft rejection became increasingly focused on unpicking the genetics of immune response, focusing on the SLA. For this, they employed some of the same comparative approaches as other workers on the genomics of the pig, using materials, methods, and maps and sequence from human genomics to aid their elucidation of the fine-grained structure and polymorphisms of the SLA.

This kind of work was important for understanding the immune response – and rejection – initiated when a transplantation took place between two individuals of the same species. Xenotransplantation entailed an extension of this, towards understanding the relevant genetic (and other) differences between pigs and humans, which are crucial to understanding how the human body can reject organs that come from pigs. In the oft-cited words of xenotransplantation researcher Claus Hammer, in order to overcome the genomic, immunological and physiological divergences between human and pig since their last common ancestor approximately 80 million years ago, we need to “outwit evolution” (Hammer, 1998, p. 26). In other words, xenotransplantation needs to overcome some of the panoply of changes that have occurred in the genomes of both lineages in the intervening period. The project of comparative genomics has involved tracing these changes, through interrogating the resolution and nature of particular sites of commonality between the genomes of the two species. The mapping using the RH panel was a significant step in this process, resulting in a comparative map that represented a further refinement of knowledge of re-arrangements and other forms of genomic change and evolution. This map, in turn, helped researchers to make more fine-grained examinations of genomic changes and differences between humans and pigs, some of which bear responsibility for the rejection of tissues and organs transplanted from one to the other.

Unlike islet cells and heart valves that are invisible to the immune system of the host, highly-vascularised organs cannot be isolated from the immune system, so rejection is a problem that must be tackled.^31^ The problem of hyperacute rejection has been solved. This involves knocking out (inactivating) a gene called *GGTA1*. The gene codes for an enzyme called α1,3-galactosyltransferase, which in the late-1980s was found to be present in many mammals, with the exception of humans, other apes and Old World monkeys (these include macaques that are used in xenotransplantation studies; Galili et al., 1988). This enzyme catalyses the addition of a chemical residue to produce the immunoreactive α-galactosyl epitope – the part of an antigen that is recognised by the immune system – on the surface of pig endothelial cells that line the inner surfaces of blood and lymph vessels.

Further investigation in the early-1990s demonstrated that the human antibody anti-Gal specifically binds to this epitope in pigs, leading to hyperacute rejection within minutes due to the cells bearing the epitope being attacked and destroyed by the host immune system. This finding led to a collaborative team – including researchers from the UK Institute for Child Health and the Department of Animal Breeding and Genetics at the Swedish University of Agricultural Sciences – mapping the *GGTA1* gene in pigs in 1994. They used a bovine DNA probe to screen a pig DNA library, given that cattle also express the epitope. Sequencing of the relevant pig DNA identified this way, allowed the researchers to make comparisons with other mammals expressing the epitope.^32^ Once they established the chromosomal location of the gene, they were able to identify the corresponding homologous region of the human genome. There they found a degraded homologue of the *GGTA1* gene (a pseudogene), supporting the hypothesis that the gene had become inactivated in the lineage leading to humans (Strahan et al., 1995).

Subsequent studies augmented the genomic data concerning this gene, and strategies to inactivate the gene began to be developed. This culminated in the genetic modification of pig foetal cells, the selection of those cell lines in which the gene had been successfully knocked out, and then nuclear transfer into oocytes using the techniques developed to clone Dolly the sheep (Lai et al., 2002). Lines of animals could therefore be produced with the *GGTA1* gene reliably knocked out. This ensured that the part of the enzyme that makes it work the way it does is removed and therefore that the molecular red flag to the human immune system – the xenoantigen – was no longer produced.

The development of these knockout lines enabled the worst hyperacute rejection to be dealt with. A variety of other genetic and immunological differences between pigs and humans that cause rejection on slightly longer timescales have been uncovered. These differences include both antigens produced by the pig, and complement regulators in the human (Sykes & Sachs, 2019). Complement regulators help the immune system to distinguish between self and non-self. By inserting human complement regulators into the pig genome, the human immune system can be tricked into perceiving that the cells are human and not pig. One approach has been to stack multiple inserted genes at the same site in the pig genome, with the location chosen on the basis that the addition of these genes there would not disrupt other genes (Fischer et al., 2016). The advent of genome editing has allowed the deletion of PERVs. It has also enabled the well-targeted and more efficient deletion of stretches of DNA to deactivate genes responsible for the production of pig epitopes that generate a human immune response (e.g. Cooper et al., 2019). As a result of these measures, the pig genome and immune system therefore slowly become humanised, as pig-specific genes are inactivated (they become pseudogenes like some of their human homologues) and human genes are transferred into the pig genome.

However, it is one thing to successfully genetically edit and modify pigs to ensure that the organs will not be immediately rejected by the human recipient. It is quite another to ensure that the organs are in fact transplanted successfully, that the recipients survive, and that longer-term chronic rejection is avoided. Here, the time scales become longer, and the underlying biology becomes much more complicated. Pig organs are eventually rejected in the non-human primates into which they are experimentally transplanted, and these rejections must be reverse engineered to understand what further genetic and physiological processes are undermining acceptance. The same investigations will take place following the transplantation of pig organs into humans (Reardon, 2022).

Genomics has been significant, in enabling us to sequence DNA, to catalogue variants, and to identify precisely which bits of DNA to edit. It has also helped make it increasingly evident that to properly understand any aspect of biology, one must understand that not just one or a few genes are involved, but many, interacting in ways that are difficult to discern (e.g. Boyle et al., 2017), in addition to ongoing dialogue between the organism and its environment (Fox Keller, 2015).

### Humanisation and dehumanisation in xenotransplantation and comparative genomics

For the humanisation of pigs required for xenotransplantation, the development and exploitation of inferential models of biological correspondence (genetic/genomic, physiological, immunological) must be pursued and continually refined. This itself uses established genomic resources on the pig that were developed through managing the tension between humanisation and dehumanisation described in Sect. 2. It is no good, however, if the models for xenotransplantation are based on genomic resources for the pig that have been humanised, as the point is to find genetic and immunological points of difference that can be engineered away. Thus, the concerns raised by the physical mappers and Alan Archibald cited in Sect. 2.2 about the potential humanisation of the pig genome (through the use of human genome data and materials) have a practical salience beyond scientific rigour.

In the case of genomics, transposition and the role of models of correspondence in mediating between two species, pertain to *representations* of those species. These representations take the form of maps, databases, and bodies of knowledge concerning the differences and similarities between genomes.^33^ They also include the biology of genome evolution that helps to explain and make sense of them. As previously explored, comparative genomic work provides a basis for the identification of forms of similarity and difference. For transplantation biology, the pertinent comparators are the particular genes and genetic variants that result in the production of biological molecules (such as antigens) implicated in immune response, and therefore forms of rejection.

Whereas in comparative genomics, the transposition between genomes is in the representative mode, in xenotransplantation, transpositions between species are intended to be *material*. The models of correspondence and processes of transposition are intended to help successfully transfer an organ or tissue from a pig to a human. This shift in the object of transposition or mediation alters the epistemic aims or goals of the exercise, and therefore the nature of the epistemic iterativity that manifests. The improvement of the precision and accuracy of representations of the genome in comparative genomics constitutes an open-ended form of iterativity in which improvement is discerned in several respects. One is with reference to a separate domain of equivalent epistemic iterativity that is taken – with justification – to be more advanced: human genomics. Others operate more internally to pig genomics, rather than to any external standard. These rely on triangulating independent sources of data, and generating and using metrics that compare newer representations with older ones.^34^

In xenotransplantation, however, the standard by which improvement is measured is based on the timescales and severities of rejection. There is, therefore, a more-or-less objective external benchmark by which the iterativity can be assessed and further advanced. A new gene deletion or insertion is tested by the survival times of the organ recipients. If it is deemed to be successful by that benchmark, the reasons for the eventual failure of the transplant can be identified, debated and tested, and new alterations proposed and evaluated. However scientifically and technically complex the whole endeavour is, the epistemic iterativity is simplified: it is focused on a specific goal, and only some of the genome is identified as relevant and therefore targeted. This has the implication that, by contrast to comparative genomics, (xeno)transplantation biologists can be less concerned with the risk of humanisation, as their narrower and function-oriented focus means that they are concerned with understanding – and potentially amending – only small parts of the genome. Humanisation is necessary, but also necessarily limited. For the comparative genomicists, however, for whom the whole genome is their canvas and on which the activities of practice-oriented scientists rely, both the consequences and the risks of allowing humanisation to find its way into their representations would be grave.

## Conclusion

The speed and scale of research on the human genome enabled the pig genomics community to adopt and adapt tools, methods and organisational approaches for the genomic mapping (and later sequencing) of their own species. They were able to harness the genomic conservation that they increasingly appreciated, to use the considerable amounts of data and materials produced by human genomics in their own work. Through devising and executing a variety of ‘trans-species shuttling strategies’, such as the use of human ESTs and mapping data in radiation hybrid mapping and later the exploitation of human DNA sequence data, models of correspondence between the respective genomes of pig and human were constructed and continually refined.

The data, resources and models produced through trans-species shuttling strategies were used in both MHC research concerning allotransplantation, and the connected programme of research aiming to enable the transplantation of organs from pigs to humans. The latter has used genomic resources on the pig to: identify and delete PERVs from pig genomes and evaluate the wider effects of this editing; identify, edit and validate the removal of genes like *GGTA1*; and add human complement regulators by transgenic methods.

The humanisation of pigs can be effected by genome editing, transgenics, the production of synthetic porcine genomes, or the creation of chimeras containing human cells, tissues and organs in the body of a pig. In all cases, the re-engineering of the pig can also be used to produce new biomedical models. In Davies’ discussion of humanised mice produced for biomedical research (2012), she describes the delicate balance required, of the humanised mouse needing to be “mouse enough,” not too mouse, not too human. This is indeed the case in xenotransplantation. It is not, however, in comparative genomics, where the representations of the pig genome cannot simply be ‘pig enough’. There is no balance to be struck here: any residues of humanisation that are left by the process of using representations, tools and materials from human genomics must be washed out in the process of strictly monitoring, maintaining and reinforcing species boundaries. This distinction between the domains of genomics and xenotransplantation masks a deeper connection between the two fields, as the straddling of boundaries manifested in xenotransplantation depends on the assiduous attention to boundaries in comparative genomics.

Dam et al. (2018) have pointed to a tension concerning boundaries in translational research that used preterm (premature) piglets to study a potentially-fatal gut disease (necrotizing enterocolitis) that can affect preterm babies. Here, the translational demands to make the piglets more like the babies, that is, to encourage cross-species “contamination”, threatened the “moral demarcation” between species that enabled the piglets to be used as models in this way, entailing a “continuous spatial containment” to keep the pigs and the humans properly demarcated. While I have only discussed technical aspects of xenotransplantation, there are undoubtedly ethical, moral and legal reasons for containment and demarcation here as well, in addition to qualms about the potential for inter-species hybridity.

The tension between humanisation and dehumanisation that I have explicated in this paper to make sense of the dynamics of comparative genomics and xenotransplantation research are of potential relevance beyond the cases described. Comparative strategies similar to the ones articulated in this paper have been used between humans and other mammals (e.g. humans and mice), between non-human mammals (e.g. hamsters and pigs) and even between organisms in different higher-level taxonomic groups (e.g. chickens and humans). Examining some of these may help us to further appreciate the wider infrastructural role of human genomics in genomics research concerning non-human species.^35^ Furthermore, once the genomic resources and data pertaining to a given species has reached a certain level of resolution and stability, these may in turn be used to seed comparative approaches to develop analogical models within species, for example between given sub-specific populations (e.g. for the pig, see Groenen, 2016; Yang et al., 2017).

Exhortations to decentre humans (and sometimes, animals more broadly) as an archetype or benchmark have grown in historical, philosophical and social scientific studies of biological research. These include the characterisation of evolutionary and genetic processes from the perspective of microorganisms rather than metazoans (O’Malley, 2014), the articulation of the holobiont in place of traditional conceptions of biological individuality (Pradeu, 2016), centring the role of animals and other non-humans as historical agents (Woods et al., 2017), and shifting the depiction and analysis of genomics research away from narratives dominated by human genomics and the human genome project (García-Sancho & Lowe, Forthcoming).

In some of these examples, humans and human-centred objects and endeavours remain pertinent. In these, the scholarly dehumanisation must therefore operate in tandem with a degree of humanisation. Comprehending the development of non-human genomics, and other endeavours in which human-centred research has affected work on other species and objects, demands that we focus attention on the ongoing construction of nexuses between the human and the non-human. This embedding of the human in constructed and evolving relationships with other organisms will help to ensure that decentring the human does not lead to human exceptionalism being reproduced in an inverse way.

## Footnotes

1 The world’s first pig-to-human heart transplant has now been performed, but at the time of writing considerable work remains until the xenotransplantation of hearts and other organs can become a routine clinical procedure (Reardon, 2022).

2 Other proposals for humanising the pig include creating inter-species chimeras by implanting human stem cells into the early embryo of the pig, to enable these cells to develop into particular organs (Waltz, 2017). This confronts far greater scientific and technical obstacles than for xenotransplantation.

3 These efforts had meagre origins, e.g. 42 genes or genetic markers assigned to loci as of 1991 (O’Brien, 1991). This sparsity of mapped genes and genetic markers was not unique to the pig. In 1991, only two mammals (human and mouse) had thousands mapped, and only rats had hundreds mapped (O’Brien, 1991). Markers, or genetic markers, are any kind of DNA sequence variation that can be detected and localised in some way in a chromosome. The mapping of genetic markers was particularly significant in livestock genetics and genomics (Lowe & Bruce, 2019).

4 Pig Gene Mapping Project (PiGMaP). Proposal for grant from European Commission’s BRIDGE programme. Obtained from Alan Archibald’s personal archive, Roslin Institute, Scotland, on 24th March 2017.

5 This drew on existing relationships that pig genome researchers had with human genomics and human genome projects, such as the Medical Research Council’s Human Genome Mapping Project. This gave pig genome researchers an acute awareness of the human genome resources available to them, influenced the nomenclature and data sharing practices they adopted (Archibald et al., 2010; Hu et al., 2011), and encouraged them to use bacterial artificial chromosomes in the construction of their genome libraries (Lowe, 2018, pp. 6–7).

6 The need to foster new standards, norms, infrastructures and moral economies to further human genome mapping has been elaborated by Bostanci (2004) and Hilgartner (2017).

7 There was also a tradition of comparative sequence (e.g. DNA and amino acid) analysis dating back to the 1950s (Dietrich, 1998; Strasser, 2019; Suárez-Díaz, 2014). While significant, it does not play a key role in the modes of research examined in this paper, which are better understood as part of other – e.g. quantitative and molecular genetic and immunogenetic – historical lineages.

8 These fragments, from a now-dead donor cell of the organism you wish to map, are hybridised to recipient cells of another species (Cox et al., 1990 used Chinese hamsters). To detect the presence or absence of known markers in the fragments, Southern blotting is performed, in which the DNA is digested by restriction enzymes and then undergoes gel electrophoresis. It is then transferred to a membrane that is treated with labelled DNA probes that are intended to hybridise to the donor DNA. Originally radioactive probes were used and the results of the gel electrophoresis were captured by autoradiograph, but fluorescent probes traced with photographs were used from the mid-1990s onwards.

9 This was version 22 of the human genome assembly.

10 The ‘N’ was for Nevada.

11 The statistical threshold levels are a matter of norms and judgement, and the possibility of spurious correlations using BLAST is a potential problem, especially when the alignments are not otherwise supported by other genomic and cytogenetic data.

12 In the BAC libraries, 297,742 BES with at least 100 bases of non-repetitive sequence and sufficient alignment to human sequence were identified using BLAST, 48% of the total.

13 This was done by digesting the clones with a restriction enzyme (*Hin*dIII) and then running the fragments through gel electrophoresis.

14 Alan Archibald, interview with author, Roslin Institute, Scotland, 17th November 2016.

15 In her analysis of *C. elegans* genome mapping, de Chadarevian (2004) has made a similar point. She observes that the production of new kinds of maps, rather than representing a displacement of prior kinds, was driven by concrete demands often independent of those that drove the construction of preceding maps. These newer maps were not wholly independent though, as they built on preceding maps, and in turn helped enrich these earlier ones as resources, in part because of the infrastructuring work (after Star & Bowker 2002) that enabled each map to be commensurated with others.

16 As noted above, I derive the terms “material” and “intellectual” abstraction from Leonelli (2008).

17 Alan Archibald, interview with author, Roslin Institute, Scotland, 17th November 2016.

18 We may also note a wider iterativity beyond a dyad of two species. As observed by Mason Dentinger & Woods (2018; see also Hagen 2018) in a different context, “comparison was an iterative process: once a basis for comparison between two species was established, it often became a conduit by which increasing numbers of species or increasing numbers of organismic qualities could be justifiably compared.”

19 Symmetrical in the sense that it enables transposition in multiple directions, rather than in the sense of the direct equivalence of parts across species articulated by Franklin (2006) and used in Davies (2012).

20 Other significant pig genomics groups have also been explored in the literature (Lowe, 2018, 2021; Lowe et al., 2022). Other researchers have been far more involved in the practical aspects of xenotransplantation research, such as David Cooper, David Sachs and Angelika Schnieke.

21 Nevertheless, in understanding mechanisms of rejection and in effecting improved survival times, transplants from pigs to non-human primates have been performed en route to clinical testing, due to the greater physiological and genetic proximity of non-human primates to humans.

22 https://web.archive.org/web/20170723073859/https://www.pbs.org/wgbh/pages/frontline/shows/organfarm/business/business.html (accessed 12th October 2022).

23 Unless otherwise specified, the sources for this section come from an interview I conducted with Patrick Chardon, Christine Renard and Marcel Vaiman, in the presence of Claire Rogel-Gaillard, in Paris, 28th November 2017. The institution went through several names, and was mostly known as Laboratoire de Radiobiologie Appliquée (Laboratory of Applied Radiobiology) and Laboratoire de Radiobiologie et Etude du Génome (Laboratory of Radiobiology and Genome Study). I refer to it as CEA-INRA to provide a simple fixed name.

24 The immediate prompt for this was the unsuccessful bone marrow transplantation treatment of people who had lost their bone marrow due to exposure to whole-body gamma radiation, leading to the CEA funding Marcel Vaiman. The role of atomic and radiation research in promoting and controlling new lines of (radio)biological research has been well-documented, e.g. Adamson (2009), de Chadarevian (2006), Creager (2013), Gaudillière (2006) and von Schwerin (2013).

25 Examples of this collaboration in its nascent stage were the help given to Christine Renard – a key member of the laboratory from the 1960s to the 2000s – to adapt a computer programme to study antibodies, and the development of serological methods to map the SLA, which Dausset requested of Vaiman. The group working on the HLA had encountered problems that they deemed to be more tractable in the pig, due to the ability to create large families and selectively cross pigs, something not possible for human genetic analysis. A UK team also demonstrated the existence of a porcine MHC contemporaneously (Viza et al., 1970).

26 The number of alleles and haplotypes revealed by CEA-INRA reagents in serotyping were very high: 63 different haplotypes, covering many breeds. The problem is that for a serological system, reagents are required to type an animal for new breeds, and the right reagents may not be available. This is why Patrick Chardon, a member of the CEA-INRA team, suggested to me that DNA typing was more efficient.

27 In ‘EC PiGMaP progress report final and individual labs – 1992’ file, obtained from Alan Archibald’s personal archive, 24th March 2017.

28 In ‘5th EC PiGMaP Meeting 1994 Edinburgh full report’ file, obtained from Alan Archibald’s personal archive, 24th March 2017.

29 The BAC library was produced using DNA from a Large White pig, genetically homozygous for the SLA system.

30 Alternative stages denoting particular mechanisms of immune rejection are also used, but the basic temporal categories are more appropriate for this discussion.

31 Pig heart valves have been transplanted into humans for decades. More recently, islet cell transplants into the pancreas to treat diabetes have been achieved. These, however, have the benefit of being relatively immunologically inert. For the heart valves this is due to them being treated with a chemical called glutaraldehyde, for the islet cells this is because they are encapsulated in a special polymer. This lets oxygen, nutrients, insulin, and waste in and out, but prevents detection by the host’s immune system. Such encapsulation has been suggested as a reason why PERVs have not infected the body after test transplants (Denner et al., 2018).

32 The DNA in the probes and the library was complementary DNA (cDNA) derived from messenger RNA, rather than being DNA directly obtained from the genome.

33 The way that such difference is ascertained and measured depends on the comparator objects chosen: whole-genome DNA sequences; selected parts of the genome known to have particular rates of mutation; the nucleotides that comprise genes; or the amino-acid sequences of proteins. The choice of comparator relates to the purpose of comparison, such as: identifying evolutionary relationships between species; discerning the timings of evolutionary processes such as the formation of separate lineages from their most recent common ancestor; or identifying differences that may pertain to structural and functional distinctions.

34 For a discussion of evaluative methods in genomics, see García-Sancho & Lowe (Forthcoming).

35 For an in-depth demonstration of the infrastructural nature of “reference resources” in genomics, and the infrastructural role that this gives genomics itself in the wider life sciences, see García-Sancho & Lowe (Forthcoming).
